# Repeat late instent-stenosis after an interval of four years in the same lesion after bare-metal and drug-eluting stent: a case report

**DOI:** 10.1186/1757-1626-2-9407

**Published:** 2009-12-31

**Authors:** Frank Towae, Ralf Zahn, Uwe Zeymer

**Affiliations:** 1 Department of Cardiology Herzzentrum Ludwigshafen, Medizinische Klinik B, 67063 Ludwigshafen, Germany

## Abstract

In 2001, a 71-year old male was admitted to our hospital with unstable angina. The angiography revealed 2-vessel disease with a 90% stenosis of the proximal LAD. A bare-metal stent was implanted. Four years later the angiography showed a 80% instent-stenosis in the bare-metal stent but no progress at the other coronary arteries. A DES was implanted. Again, four years later, the patient presented with non-ST-elevation myocardial infarction. Angiography showed a 90% instent-restenosis, again without any progession of coronary artery disease in the other vessels. Again a DES implanted. Therefore the processes involved in the late instent-stenosis were not influenced by the antiproliferative agent sirolimus

## Case presentation

**Figure 1:**
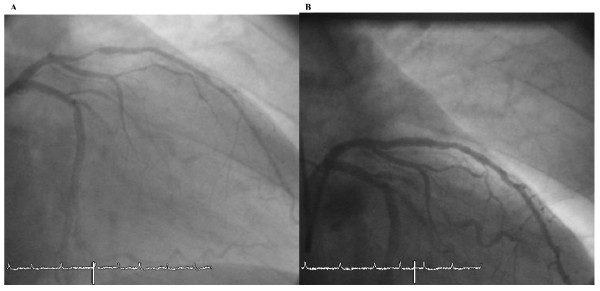
**Angiography after implantation of a bare-metal stent (2001)**.

**Figure 2:**
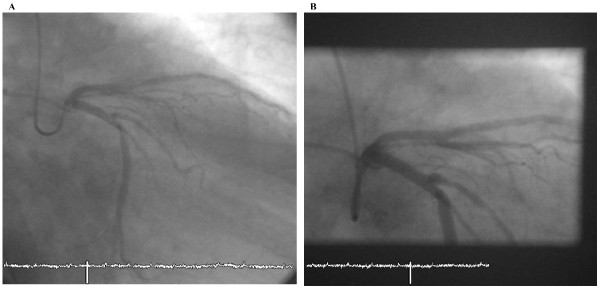
**Second instent-stenosis in 2009**.

**Figure 3:**
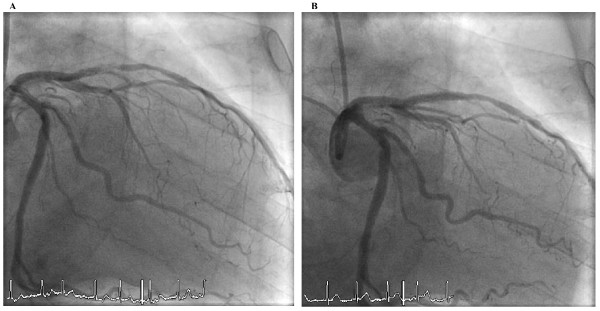
**A. Second instent-stenosis in 2009**. b. Final result after PCI with drug-eluting stent in 2009.

In April 2001 a 71-year old male (German) was admitted to our hospital with unstable angina. Blood pressure at admission was 160/80 mmHg, LDL cholesterol was 127 mg/dl and no diabetes exist. The electrocardiogram showed persistent atrial fibrillation with negative T-waves in leads V_2_-V_5_. The angiography revealed 2-vessel disease with a 90% stenosis of the proximal LAD (Figure 1a). A bare-metal stent (3.0/18 mm) was implanted (Figure 1b). Aspirin und clopidogrel were prescribed against stent-thrombosis by the use of bare-metal stent for 4 weeks followed by oral anticoagulation with a vitamin-K antagonist (INR 2-3, phenprocoumon). Four years later the same patient was hospitalized once again with stable angina. Blood pressure was 130/70 mmHg at admission and in the meanwhile the patient had diabetes. The angiography showed a 80% instent-stenosis in the bare-metal stent but no progress at the other coronary arteries (Figure 2a). This time a sirolimus-eluting stent (CYPHER 3.0/23 mm) was implanted (Figure 2b). Aspirin, clopidogrel and the same oral anticoagulation (phenprocoumon) were given for three months against stent-thrombosis by use of drug-eluting stent followed by oral anticoagulation alone. Again, four years later, in February 2009, the patient presented with non-ST-elevation myocardial infarction. Blood pressure was 120/80 mmHg at admission and the patient presented with the same risk factors as prescribed. Angiography showed a 90% instent-restenosis in the LAD again without any progession of coronary artery disease in the other vessels (Figure 3a). Again, a drug-eluting stent (XIENCE V 3.0/28 mm) implanted (Figure 3b).

## Discussion

This case is unusual, because a late instent-stenosis was observed twice without any difference in the time interval of the clinical occurrence of the restenosis between bare-metal and drug-eluting stent [1-3]. Usually restenosis occurs within 6 months after stent implantation. Thereafter the annual restenosis rate is only about 1-2%. In this case, the implantation of a DES did not prolong the time-interval until the restenosis became clinically apparent. Therefore, the processes involved in the late instent-stenosis in this patient were not influenced by the antiproliferative agent sirolimus [4,5].

## Consent

Written informed consent was obtained from the patient for publication of this case report and accompanying images. A copy of the written consent is available for review by the Editor-in-Chief of this journal.

## Competing interests

The authors declare that they have no competing interests.

## Authors' contributions

FT collected the data and wrote the case report. RZ reviewed the report and gave suggestions. UZ reviewed the report and gave suggestions.

All authors have read and approved the final manuscript.
